# The effect of carbonic anhydrase IX on focal contacts during cell spreading and migration

**DOI:** 10.3389/fphys.2013.00271

**Published:** 2013-10-01

**Authors:** Lucia Csaderova, Michaela Debreova, Peter Radvak, Matej Stano, Magdalena Vrestiakova, Juraj Kopacek, Silvia Pastorekova, Eliska Svastova

**Affiliations:** ^1^Department of Molecular Medicine, Institute of Virology, Slovak Academy of SciencesBratislava, Slovakia; ^2^Centre for Molecular Medicine, Slovak Academy of SciencesBratislava, Slovakia; ^3^Laboratory of Bioinformatics, Institute of Molecular Biology, Slovak Academy of SciencesBratislava, Slovakia; ^4^BioScience Slovakia s.r.oBratislava, Slovak Republic

**Keywords:** carbonic anhydrase IX, focal adhesion, migration, paxillin, cell spreading, ROCK1

## Abstract

Carbonic anhydrase IX is a hypoxia-induced transmembrane enzyme linked with solid tumors. It catalyzes the reversible hydration of CO_2_ providing bicarbonate ions for intracellular neutralization and protons for extracellular acidosis, thereby supporting tumor cell survival and invasiveness. CA IX is the only human CA isoform containing the proteoglycan (PG) domain in its extracellular part. The PG domain appears to enhance the catalytic activity of CA IX and mediate its binding to the extracellular matrix. Moreover, manipulation of the CA IX level by siRNA or overexpression modulates cell adhesion pathway so that in the presence of CA IX, cells display an increased rate of adhesion and spreading. Here we show that deletion of the PG domain as well as treatment with the PG-binding monoclonal antibody M75 can impair this CA IX effect. Accordingly, CA IX-expressing cells show more prominent and elongated maturing paxillin-stained focal contacts (FC) than CA IX-negative controls, proving the role of CA IX in cell spreading. However, during active cell movement, CA IX is relocalized to lamellipodia and improves migration via its catalytic domain. Thus, we examined the influence of CA IX on FC turnover in these structures. While the lamellipodial regions lacking CA IX display dash-like adhesions, the CA IX-enriched neighboring regions exhibit dynamic dot-like FCs. These results suggest that CA IX can promote initial adhesion through its PG domain, but at the same time it facilitates formation of nascent adhesions at the leading edge of moving cells. Thereby it may allow for transmission of large forces and enhanced migration rate, presumably through catalytic activity and impact of pHe on FC dynamics. Thus, we provide the first evidence that CA IX protein localizes directly in focal adhesion (FA) structures and propose its functional relationship with the proteins involved in the regulation of FC turnover and maturation.

## Introduction

The process of focal adhesion (FA) is a necessary step accompanying the migration and invasion of tumor cells during their metastatic dissemination. The adhesome, a network of multiprotein complexes that coordinates FA signaling, includes at least 180 proteins (Zaidel-Bar and Geiger, 2010). Carbonic anhydrase IX is structurally predisposed to participate in this network. It is a cell surface protein consisting of the extracellularly exposed proteoglycan-like region (PG) and catalytic (CA) domain, which is anchored in the plasma membrane by single-pass transmembrane region and short intracytoplasmic tail (IC). Protein kinase A phosphorylates threonine in the IC tail of CA IX and regulates its enzymatic activity (Ditte et al., 2011). The PG-domain is a unique feature of CA IX as it is present only in this isoform of all known carbonic anhydrases (Opavsky et al., 1996). Full-length PG domain exhibits 38% homology with a keratan-sulfate region of aggrecan, a cartilage protein which can interact with ECM components hyaluronan and collagen (Opavsky et al., 1996; Nishimura et al., 1998; Hedlund et al., 1999). This similarity between CA IX and aggrecan indicates possible CA IX involvement in cell-ECM interactions. Moreover, the FA process is also influenced by the extracellular pH (pHe) which is, among other proteins, also modulated by CA IX via its enzymatic activity (Svastova et al., 2004; Stock et al., 2005; Ludwig et al., 2013).

CA IX is expressed in many solid tumors, though lacking in the corresponding normal counterparts (Pastorekova et al., 2006). In different tumor types, CA IX is considered a clinically useful biomarker with prognostic and/or predictive values and as a therapy target. Particularly high and frequent expression of CA IX is present in renal cell carcinomas, where the CA IX-specific monoclonal antibody G250 is evaluated as a promising tool for immunotherapy (Oosterwijk-Wakka et al., 2013). *CA9* gene is strongly regulated by hypoxia as a direct target of the hypoxia-inducible transcription factor (HIF-1) binding to its core promoter (Wykoff et al., 2000). Hypoxic tumors are among the most aggressive ones as hypoxia leads to microenvironmental changes, such as acidosis and lack of nutrients, which promote the development of promigratory and proinvasive cell phenotype (Chiche et al., 2010). Hypoxia is also functionally linked to altered matrix properties (Erler and Weaver, 2009) through e.g., upregulation of collagen synthesis and remodeling of the ECM by prolyl 4-hydroxylase (P4H) and lysyloxidase (LOX) (Fahling et al., 2004; Postovit et al., 2008). Extracellular acidosis enhances the activity of matrix metalloproteases (MMP) either directly by protonating them or their substrates or indirectly by affecting their exocytosis (Holman et al., 1999; Monaco et al., 2007; Iessi et al., 2008). All these hypoxia-induced changes of the extracellular matrix and pHe facilitate escape of tumor cells from hostile conditions.

CA IX is well-known for its role in pH regulation and acidification of tumor microenvironment, which is based on its ability to catalyze conversion of CO_2_ to H^+^ and HCO^−^_3_. The underlying mechanism includes CA IX-generated bicarbonate ions that directly feed bicarbonate transporters for the neutralization of intracellular pH (Swietach et al., 2009; Orlowski et al., 2012). On the other hand, simultaneously produced protons support extracellular acidosis, particularly in hypoxic tumors (Svastova et al., 2004). We recently proved the importance of the catalytic activity of CA IX for the enhancement of cell migration and direct interaction of CA IX with the bicarbonate transporters NBCe1 and AE2 in migratory organelles known as lamellipodia (Svastova et al., 2012). Interestingly, several proteins involved in the adhesome are either pH sensors and/or their activity is influenced by pH (Srivastava et al., 2007; Stock and Schwab, 2009).

The formation and strength of FA are also influenced by the extracellular (pHe) and intracellular pH (pHi) (Lehenkari and Horton, 1999; Stock et al., 2005; Srivastava et al., 2008; Paradise et al., 2011). Assembly of FA sites is a gradual process requiring the step-by-step recruitment of individual proteins that connect integrins and other ECM receptors with actin cytoskeleton. Integrins recruit adaptor and signaling proteins, such as paxillin, vinculin, talin, focal adhesion kinase (FAK), Rho GTPases, etc. (Webb et al., 2002; Parsons, 2003). Focal contacts (FCs) grow and dissolve in close relation to actin polymerization and myosin II-generated tension (Vicente-Manzanares et al., 2009). A central molecule for both assembly and turnover of FCs is the adaptor protein paxillin, which directly binds to integrins (Zaidel-Bar et al., 2007). It can also recruit FAK into an adhesion plaque and trigger its autophosphorylation at Tyr397 which creates a binding site for Src family kinases (Worth and Parsons, 2008). This leads to further FAK phosphorylation at other residues to attain the maximal kinase activity. RhoA-associated protein kinase (ROCK) is essential for myosin II-generated tension and represents a key mechanism of FA maturation. Specific inhibition of ROCK1 or downregulation of the myosin II activity decreases the size of FAs (Rottner et al., 1999; Pasapera et al., 2010). It is therefore interesting that microarray results with HT1080 cells silenced for CA IX showed approximately 50% downregulation of ROCK1 accompanied with the inhibition of FA pathway (Radvak et al., 2013).

FA in migrating cells differs from that in quiescent cells. The migratory cycle consisting of the repetitive adhesion-deadhesion of the front and rear ends of moving cells requires dynamic assembly and disassembly of FCs (Webb et al., 2002). Arising FCs in the leading edge of the lamellipodium undergo the maturation process from new, nascent adhesions to mature FAs. They differ in cellular localization, size, type of actin they are connected to, levels of individual proteins and their phosphorylation (Ridley et al., 2003; Choi et al., 2008). Nascent adhesions formed in the protruding lamellipodia contain, among other proteins, integrin receptors, talin, vinculin, paxillin, α-actinin, FAK, and are enriched in phosphotyrosine. They have a short lifespan (~60 s) and can either rapidly turnover or grow into focal complexes depending on the cell type (Parsons et al., 2010). Nascent adhesions are connected with dendritic actin and are responsible for the transmission of strong propulsive forces leading to the advancement of lamellipodia (Beningo et al., 2001). Focal complexes developing in the region located right behind the nascent adhesion area are bigger in size and can continue to mature into larger, elongated FAs anchoring thick actin bundles. Actin-crosslinking activity of the myosin II which generates acto-myosin tension, contributes considerably to adhesion maturation. Matured focal adhesions exert weaker forces sufficient for passive anchorage which are important for maintaining a spread cell morphology. Changes, to which FC are subjected during the cell migration cycle, reflect the changes of their function, strictly defined by their localization along the apical-basal axis of a migrating cell.

Here we investigated the contribution of CA IX to cell spreading and adhesion. Firstly, we showed that downregulation of the CA IX level decreases cell attachment and spreading in part by reducing expression of the ROCK1 kinase, the activity of which is critical for the myosin II-driven maturation of FA. Secondly, we provided evidence that the enhancement of CA IX-mediated cell adhesion in quiescent cells depends on the proteoglycan-domain in its extracellular part. Further, we proved that CA IX is directly localized in FC where it colocalizes with the adhesion protein paxillin from the very early stages of the cell attachment to the fully spread state. Moreover, we demonstrated that CA IX colocalizes with paxillin in nascent FAs of lamellipodia in migrating cells where the pH-modulating activity of CA IX can play a role in FC turnover. Finally, through *in silico* analysis of the gene-profiling database of clinical studies we found that different types of tumors with up-regulated *CA9* display activated FA pathway. Altogether, our findings suggest the involvement of CA IX in FA of quiescent as well as migrating cells and thereby disclose a new function for this pH-regulating, hypoxia-induced enzyme.

## Materials and methods

### Cell culture

HT1080, SiHa, HeLa, MDCK, C-33 A (C33) cells and their transfected derivates were cultured in DMEM with 10% fetal calf serum (Lonza BioWhittaker) at 37°C in humidified air with 5% CO_2_. Hypoxic experiments were performed at an anaerobic workstation (Ruskinn Technologies) in 2% O_2_, 2% H_2_, 5% CO_2_, 91% N_2_ atmosphere at 37°C.

The transduction of HT1080 cells by lentivirus system has been described elsewhere (Radvak et al., 2013). The conditional shRNA system in HT1080 cells was activated with 0.5 μg/ml doxycycline (Clontech, Mountain View, StateCA, USA) in culture medium.

Preparation of MDCK cells transfected with wt CA IX and with its deletion mutant (ΔPG) has been described previously (Svastova et al., 2004).

### Transfection

For transient silencing of *CA9* gene, HeLa cells were seeded at 1.1 × 10^6^ cells in a 6 cm Petri dish. After 4 h, cells were transfected with pSUPER-sh*CA9* to silence CA IX expression and with pSUPER-shScr as a control (Radvak et al., 2013) using TurboFect Transfection Reagent (Fermentas) according to the manufacturer's recommendations. The next day cells were trypsinized, seeded in a new 6 cm Petri dish at 9 × 10^5^ cells and incubated in hypoxic conditions (2% O_2_). Two days later cells were lysed in RIPA buffer containing a cocktail of protease inhibitors (Roche), and analyzed by western blot.

C33 cell line constitutively expressing CA IX protein was prepared by cotransfection of recombinant plasmid pSG5C-CA IX with pSV2neo in 10:1 ratio using TurboFect™ *in vitro* Transfection Reagent (Fermentas). Cells carrying an empty plasmid pSG5C were prepared and used as the negative control in cotransfection with pSV2neo. Transfected cells were subjected to selection in 900 μg/ml G418 for 2 weeks. A mixture of clones was obtained by isolation with magnetic beads (Dynabeads M-450 Tosylactivated, Invitrogen) coupled to M75 antibody specific to CA IX protein. The mixture was expanded and expression of CA IX was analyzed by FACS and immunofluorescence.

### Antibodies and reagents

For immunoblotting, protein concentrations were quantified using BCA kit (Thermo Scientific). Target proteins were detected by the following specific primary antibodies: anti-human CA IX mouse monoclonal antibody M75 in undiluted hybridoma medium (Pastorekova et al., 1992), rabbit anti ROCK1 monoclonal antibody, diluted 1:1000 (Cell Signaling Technology), goat anti β-actin polyclonal antibody, diluted 1:1000 (Santa Cruz Biotechnology). Secondary antibodies were from Sigma: goat anti-mouse IgG peroxidase-conjugated polyclonal antibody, diluted 1:8000, goat anti-rabbit IgG peroxidase-conjugated polyclonal antibody, diluted 1:12000, rabbit anti-goat IgG peroxidase-conjugated polyclonal antibody, diluted 1:5000 (Dako). The following antibodies and reagents were used for immunofluorescence: M75 antibody described above, rabbit anti-paxillin polyclonal antibody, diluted 1:250 (Santa Cruz Biotechnology), secondary antibodies from Invitrogen, diluted 1:1000: Alexa Fluor® 488 donkey anti-rabbit IgG; Alexa Fluor® 488 donkey anti-mouse IgG; Alexa Fluor® 594 goat anti-rabbit IgG, and nuclear stain DAPI (Invitrogen). Collagen was isolated from rat tails per standard procedures.

### Immunoblotting

HT1080 cells were grown for 3 days in medium containing doxycycline to induce *CA9* silencing. On the fourth day, the cells were trypsinized and seeded in a new 6 cm Petri dish at 9 × 10^5^ cells with or without doxycycline and moved to hypoxia (2% O_2_) for 48 h to induce expression of CA IX protein. The cells were then lysed in RIPA buffer containing a cocktail of protease inhibitors (Roche). Samples consisting of 80 μg total proteins were separated in 10% SDS-PAGE and immunoblotting was performed as described elsewhere (Svastova et al., 2003).

### Immunofluorescence

Cells grown on glass coverslips with or without collagen coating were fixed in 4% paraformaldehyde for 10 min and permeabilized with 0.1% Triton X-100. Non-specific binding was blocked with PBS containing 1% BSA for 30 min at 37°C. The cells were sequentially incubated with primary antibodies diluted in PBS with 0.5% BSA (PBS/BSA), each for 1 h at 37°C, washed four times with PBS containing 0.02% Tween 20 for 10 min, incubated with fluorescent secondary antibodies (always added together in one step) diluted in PBS/BSA for 1h at 37°C, washed once with PBS, incubated with DAPI (1:36 000) in PBS to stain nuclei, and then washed three times with PBS for 10 min. Finally, the cells were mounted onto slides in Fluorescent Mounting Medium (Abcam) and analyzed by Zeiss LSM 510 Meta confocal microscope in multitrack scanning mode.

### Quantification of focal adhesions in immunofluorescence assays

C33 CA IX transfected and control neo cells were seeded on collagen coated coverslips at sparse density in serum-free medium, grown for 4 h, fixed and stained as described above. Images of paxillin stained FC were taken using a Zeiss LSM 510 Meta confocal microscope at 400× magnification, zoom 3× at the same microscope settings for all samples, together with accompanying differential contrast images (DIC) to determine cell shape. The length and area of paxillin stained FCs at the cell periphery were measured using intensity thresholding in ImageJ. All measured FCs were within the designated cell area. The spreading area of cells was determined from DIC images in ImageJ software (http://rsb.info.nih.gov/ij/). Significant differences between samples were determined by *t*-test.

To quantify the degree of colocalization between CA IX and paxillin staining in FAs in HT1080 and SiHa cells during the process of initial spreading, the areas of flattened, adhered cells edges were selected using ROI tools in ImageJ software. The values of Pearson's coefficient, which measures the degree of correlative variation of the two channels in a double-stained immunofluorescence image, was calculated for these ROIs by JACoP plugin in ImageJ. Ten cells were analyzed for each phase of two cell lines used.

An analysis of lamellipodial FCs was performed on hypoxic SiHa cells. SiHa cell islands were incubated in hypoxia (2% O_2_) in DMEM with 10% FCS for 2 days. For the duration of the last 18 h the cells were starved in 0.5% FCS in DMEM and stimulated into migration by HGF (40 ng/ml) for 3 h, fixed and stained for paxillin and CA IX as detailed above. Cells with developed lamellipodia were identified, lamellipodia were dissected into CA IX—containing areas and adjacent CA IX—free areas according to CA IX fluorescent staining using ROI tools in ImageJ program. Twenty cells were used for the analysis. Analysis of FC area and length based on the intensity thresholding of paxillin staining was performed in relevant ROIs. Samples were statistically compared by *t*-test. All experiments were repeated twice.

### Cell spreading assay

MDCK and C33 transfectants and their control counterparts, respectively, were seeded on uncoated or collagen-coated coverslips in 12-well plates at the density of 100,000 cells/well and left to attach for 15 min, 30 min, 1 h, and 2 h. At the end of these time intervals, media were removed and attached cells were washed with PBS, fixed in 4% paraformaldehyde, stained with 0.5% Coomassie blue for 5 min at RT and washed 3 times with PBS. Duplicates were used in all experiments which were repeated twice. Images of the fixed cells were analyzed using ImageJ software (http://rsb.info.nih.gov/ij/). At least 200 cells were analyzed for each sample, and cell spreading areas of relevant samples were calculated and compared using *t*-test. In all cases, the mean cell diameter of trypsinized cells in suspensions was measured for various transfectants used in the same experiment by the counter (Beckman Coulter) prior to seeding. In case of different cell sizes, cell areas measured at designated times after seeding were normalized by dividing them by corresponding circular cell areas calculated using the mean cell diameter of cells in suspension. When M75 antibody was used to block the PG domain of CA IX, the procedure carried out was as follows: live cells were pre-incubated with M75 antibody (10μg/ml in DMEM with 10% FCS) for 1 h at 37°C at the rotational shaker. Cells were then washed 3 times in serum-free medium. After washing, cells were counted, seeded, fixed at indicated times and stained as described above. Control samples were subjected to the same procedure.

### Time-lapse microscopy

HT1080 cells were grown for 3 days in medium containing doxycycline to induce *CA9* silencing. On the fourth day, the cells were trypsinized, seeded, and exposed to hypoxic conditions (2% O_2_) for 48 h to induce expression of CA IX protein. Cells were then counted and seeded at sparse density into 12-well plate, in DMEM with 10% FCS, with or without 0.5 μg/ml doxycycline. Time-lapse acquisition was performed with a Zeiss Cell Observer System (Observer.Z1 microscope with motorized stage), magnification 100×, in the incubation chamber at 37°C in 21% O_2_, 5% CO_2_ atmosphere. Imaging was managed by Axiovision 4.8 software, using the Multidimensional Acquisition settings. The experiment started approximately 15 min after cell seeding (designated as time 0min), and images were taken in transmitted light every 3 min at 10 different positions for each sample. For single cell tracking analysis only unspread, round cells, very soon after their attachment to the substrate, were selected, tracked manually, and their morphometric features were calculated from time-lapse images in ImageJ program. The overall cell population, control and silenced cells, was characterized at designated times, parameters of all attached cells in the fields of view (more than 200 cells, 10 different positions for each sample) were processed and compared by *t*-test.

### Meta-analysis of gene expression profiling data

Oncomine (https://www.oncomine.org) was examined to identify microarrays datasets with significantly over-expressed CA9 (FC > 2; *p*-value < 0.0001). The Oncomine is a repository of publicly available gene expression profile data (Rhodes et al., 2007). It can be queried according to various criteria and filters. The raw data from corresponding experiments were retrieved from NCBI GEO database (http://www.ncbi.nlm.nih.gov/geo/) (Barrett et al., 2013). Statistical analysis of microarrays expression data was performed using R/Bioconductor through user-friendly graphical interface of Chipster software (http://chipster.csc.fi/) (Kallio et al., 2011). Expression data were pre-processed with an RMA procedure and filtered by flags. Differentially expressed genes between two sample groups were identified using Student's *t*-test (α = 0.05) with Benjamini and Hochberg adjustment of *p*-values. BH adjustment controls the false discovery rate (FDR) by controlling the certainty level. Sets of differentially expressed genes were subjected to Signaling Pathway Impact Analysis (SPIA) (Tarca et al., 2009). SPIA algorithm allows for the identification of KEGG pathways impacted by these differentially expressed genes. A global cut-off *p*-value was set to 0.1 in order to eliminate false discovery and at the same time preserve sufficient sensitivity of the analysis.

## Results

### Loss of CA IX decreases ROCK1 protein expression and slows down cell attachment and spreading

Our previous microarray analysis revealed the inhibition of the FA pathway in the CA IX-protein-depleted HT1080 cells (Radvak et al., 2013). In order to gain deeper insight into this phenomenon, we decided to explore one branch of this pathway with a direct impact on the cell spreading process. Indeed, the loss of CA IX resulted in a significant transcriptional down-regulation of the ROCK1 kinase, which plays an essential role in the maturation of FC during cell adhesion and spreading on the substrate as well as in the FA turnover during cell migration.

**Figure 1:**
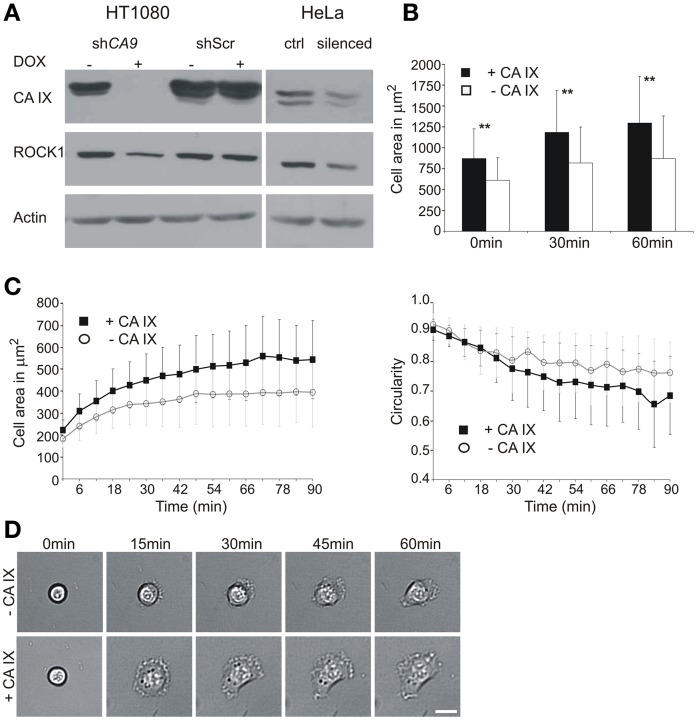
**Effect of *CA9* silencing on the expression of ROCK1 protein and initial cell adhesion and spreading**. **(A)** HT1080 cells were pre-incubated with or without doxycycline (±DOX) to induce expression of shRNA. Cells were then trypsinized and placed to hypoxia (2% O_2_) for 24 h. The level of ROCK1 protein was reduced in the *CA9*-silenced cells. Control HT1080 Scr shRNA had the same level of ROCK1 as non-silenced *CA9* shRNA (–DOX). The addition of doxycycline to HT1080 Scr shRNA cells did not cause any change in CA IX protein level, nor in ROCK1 expression. HeLa cells transiently silenced for *CA9* (pSUPER-shCA9) also showed a decreased level of ROCK1 protein compared to control cells (ctrl, pSUPER-shScr). **(B,C)** Time-lapse analysis of the entire population **(B)** and individual cells **(C)** of *CA9*-silenced and control HT1080 cells. **(B)** *CA9*-silenced cells (–CA IX) exhibited significantly smaller spreading areas than control non-silenced cells (+CA IX). Graph shows the mean ± standard deviation values for at least 200 cells, significance of differences among samples was tested by *t*-test (^**^p < 0.01). **(C)** In the analysis of individual cells, HT1080 expressing CA IX show faster spreading and achieve larger spreading area when compared with silenced cells (–CA IX). The graph of circularity demonstrates that silenced cells preserve a circular morphology for a longer amount of time, CA IX positive cells, on the opposite, became more polarized. Graphs give mean ± standard deviation values for designated time-points for 29 CA IX expressing cells and 39 silenced cells (–CA IX). **(D)** Representative pictures of characteristic morphology of *CA9*-silenced (–CA IX) and control cells (–CA IX) during the process of initial adhesion and spreading at given time-points. Scale bar 20 μm.

In line with this idea, we first analyzed protein lysates from the *CA9* mRNA-silenced HT1080 cells incubated for 24 h in hypoxia and found that these cells exhibit reduced protein levels of ROCK1 (Figure 1A). A similar decrease in ROCK1 protein expression was observed in HeLa cells with a transient *CA9* silencing, whereas no such change occurred in any of the control cells. The observation of the CA IX-related modulation of the ROCK1 protein level in both cell lines supports the functional relationship between these two proteins.

As the main function of the ROCK1-dependent pathway is to activate formation of FC through the myosin II-driven acto-myosin tension, we investigated the effect of ROCK1 downregulation, connected with *CA9*-silencing, in HT1080 cells during the process of their adhesion and spreading.

To elucidate the initial spreading process in more detail, we performed a time-lapse experiment using HT1080 cells that had been incubated in hypoxia to induce endogenous expression of CA IX and simultaneously subjected to silencing. The time-lapse experiment itself was run in normoxia for 90 min on a plastic dish, and the images were taken every 3 min, starting at the time point 0 that corresponded to ~15 min after seeding. We first compared entire populations of the *CA9*-silenced and control cells. At the beginning of the experiment (0 min) the number of control CA IX expressing cells attached to the support was higher than the number of CA IX-depleted cells and they exhibited larger spreading areas. The same tendency was observed in the later time-points (Figure 1B). These results were then confirmed by a single-cell tracking for 90 min (Figures 1C,D). From the populations we selected only the cells in the very initial stage of the spreading, shortly after their attachment and monitored them for a subsequent 90 min. At the beginning of the experiment (0 min), all selected cells were round, with the spherical shape clearly visible in the transmitted light. Throughout the course of the experiment, CA IX-expressing cells spread faster, reached larger spreading area, often became flattened, formed lamellipodia and exhibited a polarized shape (Figures 1C,D). On the other hand, CA IX-silenced cells spread more slowly, rarely formed lamellipodia and their circular morphology with a thicker central area was preserved for a longer period.

It is well-conceivable that the differences in spreading process can be attributed to CA IX-related effect on ROCK1 expression, but due to the complexity of adhesome we can expect that changes of other proteins identified in our microarray data can also contribute to the CA IX impact on FA.

### CA IX increases cell attachment and spreading in the PG-domain-dependent manner

**Figure 2:**
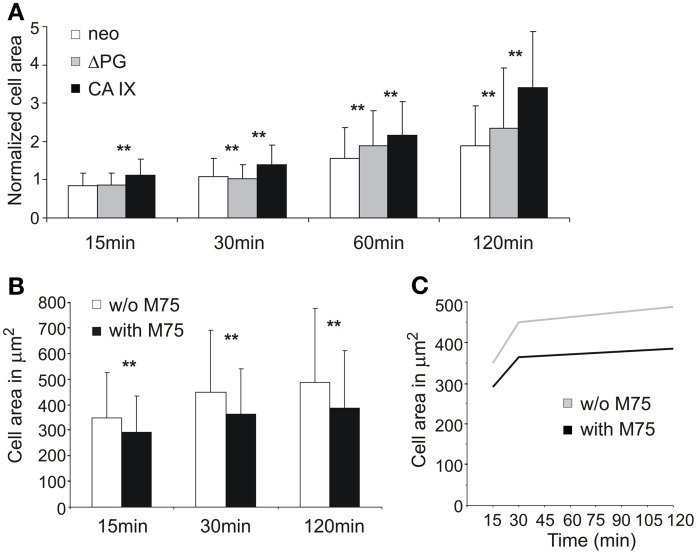
**The impact of CA IX expression on adhesion and spreading of stably transfected MDCK cells**. **(A)** MDCK cells were seeded on collagen-coated substrate and fixed at indicated time points. Expression of full length CA IX enhanced spreading area when compared with control neo cells. MDCK cells expressing Δ PG mutant decreased spreading area against cells expressing intact CA IX. Cell area was normalized to compensate for different cell sizes of used transfectant and control cell lines. **(B)** Addition of M75 antibody targeting the PG domain of CA IX led to the reduction of spreading area of adhering MDCK CA IX cells on glass coverslips. Both graphs show the mean ± standard deviation values for at least 200 cells, significance of differences among samples was tested by *t*-test (^**^p < 0.01).**(C)** Presence of M75 antibody inhibits the rate of cell spreading.

To confirm the role of CA IX in FA, we subsequently analyzed transfected MDCK cells constitutively expressing CA IX and compared them with the mock-transfected controls (Svastova et al., 2003). In the cells seeded on collagen-coated coverslips, expression of the full length CA IX protein was associated with an enhanced rate and enlarged area of spreading (Figure 2A). Interestingly, the MDCK cells ectopically expressing a deletion variant of the CA IX lacking the N-terminal PG domain spread at a similar rate to the control cells, but their spreading area was in between the areas of the full-length CA IX-expressing cells and mock controls. This observation indicated that PG domain is required for the full effect of CA IX on cell spreading. In accordance with this assumption, we found that the M75 antibody which binds to a repetitive epitope in PG domain (Zavada et al., 2000), was able to reduce both the rate and area of spreading of MDCK CA IX cells on the glass support (Figures 2B,C). These results indicate that CA IX protein can mediate the direct interaction of cells with their support.

### CA IX colocalizes with paxillin in focal contacts during initial adhesion and spreading

**Figure 3:**
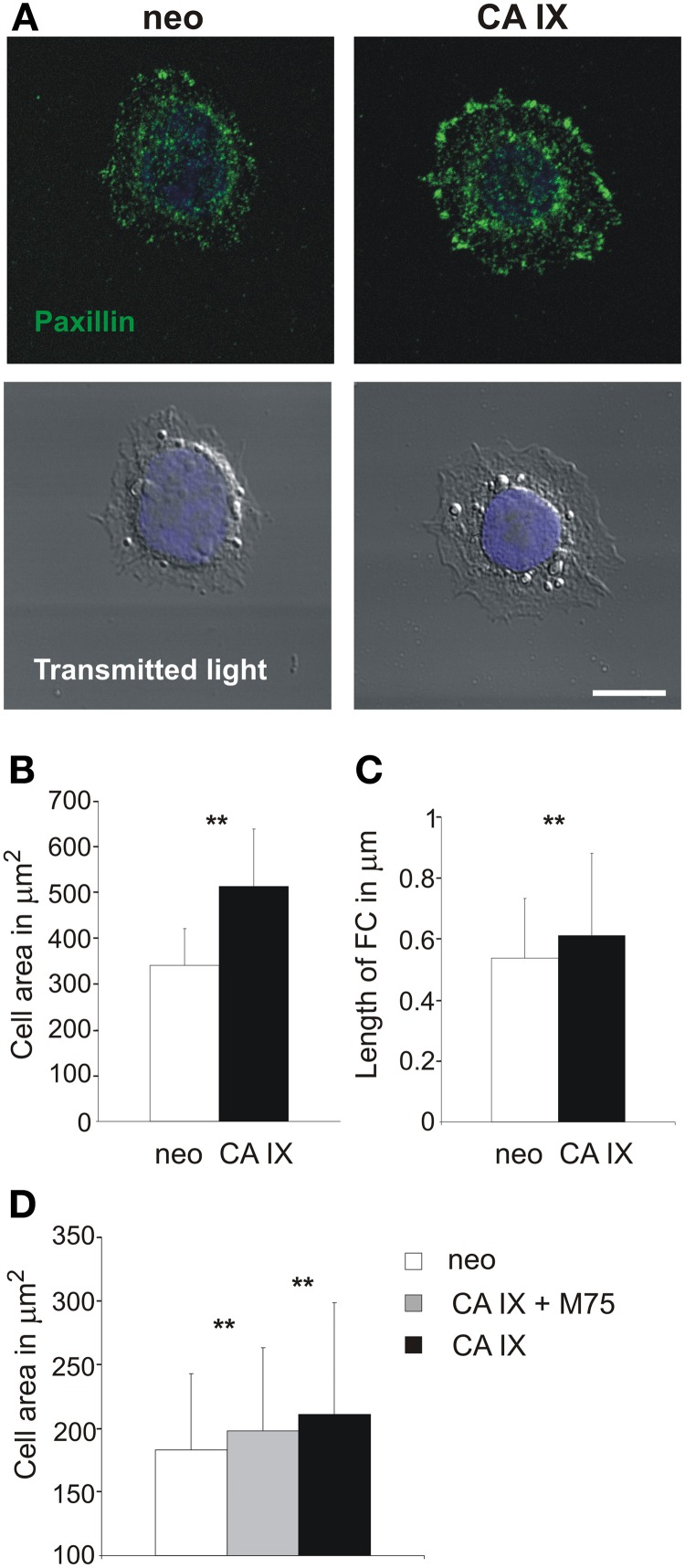
**Paxillin-based analysis of focal contacts and spreading of C33 cells with respect to CA IX expression and inhibition of PG domain**. **(A)** Immunofluorescence analysis of paxillin staining of C33 cells stably transfected with CA IX and their neo counterparts 4 h after their seeding on collagen-coated coverslips in serum-free medium. CA IX positive cells displayed larger, dash-like focal adhesions, indicating the existence of more mature focal contacts and stronger adhesion to a substrate. Scale bar 10 μm. **(B,C)** Cells were individually analyzed for spreading area, which was significantly larger for C33-CA IX cells (*n* = 20, *t*-test, ^**^p < 0.01), and also for the average length of immunofluorescently stained focal contacts (*n* = 20, *t*-test, ^**^p < 0.01). **(D)** Effect of presence of M75 antibody to PG region of CA IX on C33 transfectants. One hour after seeding on a glass coverslip, CAIX expressing C33 cells reached larger spreading areas than control neo cells, and this effect was reduced by the addition of M75 antibody. All graphs show the mean ± standard deviation values for at least 300 cells, significance of differences among samples was tested by *t*-test (^**^p < 0.01).

CA IX involvement in the process of cell adhesion and spreading was also confirmed using C33 cells transfected either with cDNA coding for CA IX or with the mock plasmid. The cells were seeded on collagen-coated coverslips and allowed to attach for 4 h. As expected, CA IX-expressing cells displayed a larger spreading area than the control cells (Figures 3A,B).

Analysis of the length and number of FAs by immunofluorescence staining of paxillin supported the conclusion of stronger adhesion of CA IX-expressing cells to collagen substrate as they displayed larger, elongated, and dash-like FAs (Figure 3C). The overall number of FCs in control neo cells was lower and FCs were smaller and more rounded (FCs per cell: 26 vs. 93 for CA IX-positive cells, *n* = 20 cells). This finding indicates that CA IX contributes to the formation of more mature and stronger contacts capable of connecting with actin stress fibers, which stabilize cells on a substrate.

To confirm the importance of PG domain for the CA IX-increased spreading, we performed the short time adhesion experiment in the presence of M75 antibody. Addition of M75 antibody suppressed the effect of CA IX on the cell spreading, similarly as in MDCK cells (Figure 3D).

**Figure 4:**
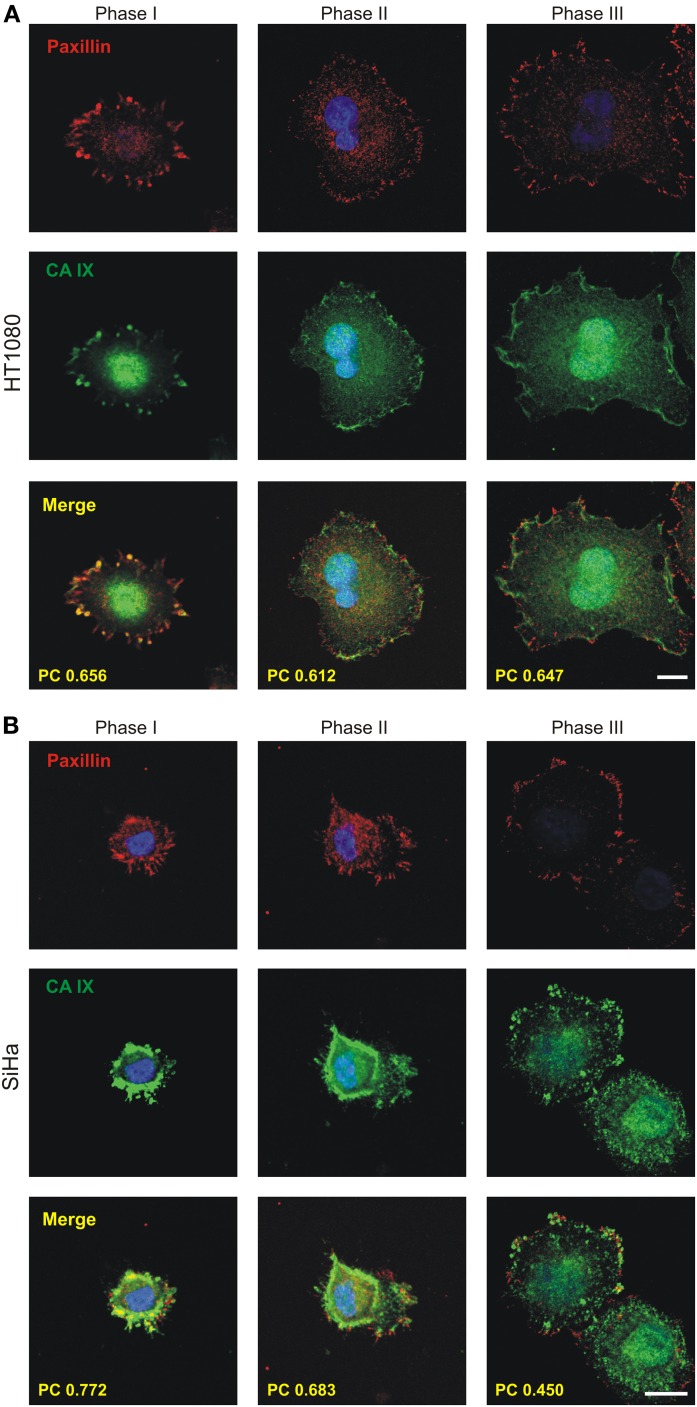
**Immunofluorescence analysis of the localization of CA IX during the process of initial spreading of HT1080 and SiHa cells with endogenous expression of CA IX**. Cells were cultivated in hypoxia (2% O_2_) for 3 days, replated on collagen-coated coverslips and fixed at different times. Samples were double immunostained for CA IX (green) and paxillin (red) and imaged by confocal microscopy. Nuclear DAPI staining is blue. Phases I-III depict cells in different time points from initiation of adhesion. During phase I, shortly after cell attachment, CA IX was accumulated at the cell periphery where focal contacts had just begun to form. In phase II, soon after cells began to spread, the colocalization of CA IX and paxillin (shown in yellow) in forming focal contacts was clearly visible. In phase III (max. 2 h after seeding), characterized by spread and flattened cells, the maturating focal contacts were represented by more elongated paxillin staining with well-preserved colocalization with CA IX. Degree of colocalization between CA IX and paxillin in each phase is expressed by Pearson's coefficient (PC) in the merged images. **(A)** HT1080 cells **(B)** SiHa cells. Scale bars 10 μm.

To characterize the CA IX localization during initial attachment and spreading, we analyzed HT1080 and SiHa cells expressing endogenous CA IX protein in response to hypoxia. The cells were first incubated for 2 days in hypoxia, then re-plated on collagen and fixed at different time points after seeding. Since HT1080 cells attached more quickly than SiHa cells, similar stages of spreading were selected and designated as phase I, II, and III instead of time indications (Figure 4). In phase I, shortly after the attachment when the cells still had a round morphology, CA IX was accumulated in the cell periphery where FCs had just begun to form. In phase II, immediately after the cells had begun the process of spreading, the colocalization of CA IX and paxillin in emergent FCs was clearly visible. In phase III, when the cells had just spread and flattened, the number of elongated dash-like FAs increased and colocalization of paxillin with CA IX was preserved. We calculated Pearson's coefficient for regions of cell edges where cells were thinned and where they had started to adhere to the surface. The values of Pearson's coefficient confirmed the high degree of colocalization for HT1080 cells in all observed phases (mean ± standard deviation values: 0.656 ± 0.049, 0.612 ± 0.045, 0.647 ± 0.052 for phases I, II, and III, respectively, *n* = 10 cells) as well as for SiHa in phases I and II (0.772 ± 0.092 and 0.683 ± 0.087, *n* = 10) and partial colocalization for phase III of SiHa cells (0.45 ± 0.16, *n* = 10). Thus, we showed that CA IX localizes in FC during the initial phases of spreading and improves cell adhesion to the substrate. Our findings imply a direct role for CA IX in these processes.

### Lamellipodial CA IX associates with sites of nascent focal contacts during cell migration

Formation, maturation, and disassembly of FAs represent a highly dynamic process which accompanies cell migration. Indeed, migratory speed is modulated by the rate of adhesion turnover and strength of FCs. As we describe here, CA IX enhances FA but on the other hand, as we described earlier it facilitates cell migration (Svastova et al., 2012). This may appear contradictory because migration is diminished in cells that are strongly attached.

**Figure 5:**
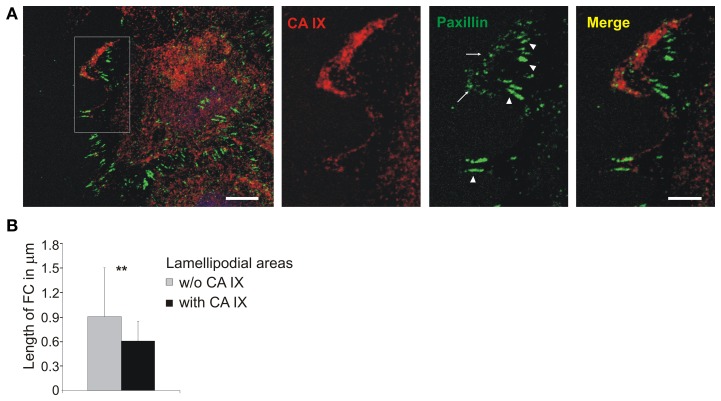
**Localization of CA IX in nascent focal adhesions in lamellipodia**. **(A)** Immunofluorescence analysis of SiHa cells grown into cell islands in hypoxia (2% O_2_), induced into migration with HGF (40 ng/ml) for 3 h. CA IX (stained red) was massively relocalized to the leading edge where it was co-stained with paxillin, a marker of focal contacts (green). The inset of a magnified lamellipodial area shows that small nascent focal adhesions were formed in the region with accumulated CA IX (arrows), whilst paxillin-stained contacts were more elongated and mature in the neighboring CA IX-free regions (arrowheads). Scale bars: 10 μm (5 μm for the inset). **(B)** Numerical analysis of area and length of focal contacts in lamellipodia from 20 cells dissected into CA IX containing regions and CA IX-free regions. Results confirmed that CA IX containing areas displayed significantly smaller and less elongated focal contacts (compared by *t*-test, ^**^p < 0.01).

In order to understand this seeming paradox, we followed the CA IX localization during cell migration in relation to paxillin staining. SiHa cells were grown into cell islands in hypoxia and then induced to migration with hepatocyte growth factor (HGF). CA IX displayed extensive redistribution to the leading edges where it was intensively stained together with paxillin (Figure 5A). Detailed analysis of lamellipodia (from 20 cells) showed that places where CA IX colocalized with paxillin were characterized by small nascent adhesions but the adjacent CA IX-free areas displayed longer, more mature paxillin contacts (Figure 5A inset). Processing of data from confocal microscope images of the lamellipodia dissected into CA IX-positive and CA IX-negative regions of interest (ROI) revealed significant differences in the pattern of paxillin contacts (Figure 5B). It is obvious that accumulation of CA IX in advancing lamellipodia occurs at the same place where cells must form transient adhesions to allow for rapid turnover of FC and thereby increase the speed of migration. Thus, the contrasting CA IX roles in FC are clearly dependent on circumstances that require either cell attachment/spreading or cell movement.

## Discussion

The maturation of FAs depends on the recruitment of adhesion proteins and their phosphorylation, matrix stiffness as well as on pericellular pH. One of the main regulators of FAs maturation is myosin II-mediated cytoskeletal tension, which regulates FAs growth, composition and phosphorylation of the constituent proteins. In turn, actomyosin-induced tension is regulated by the phosphorylation and activation of the myosin II regulatory light chain by RhoA-dependent ROCK kinases that act through inhibition of the myosin phosphatase (Vicente-Manzanares et al., 2009). Our results show that depletion of CA IX in HT1080 cells significantly decreases the protein level of ROCK1 and attenuates cell adhesiveness and spreading. This also leads to diminished staining of paxillin which is a key component of FC.

Apart from the effect on actomyosin forces, ROCK1 phosphorylates the FAK. In particular, direct FAK phosphorylation by ROCK1 on Ser732 is required for the recruitment of vinculin and subsequent maturation of the focal complex (Lock et al., 2012). Alternatively, ROCK1-induced myosin II activation can promote the FAK-mediated phosphorylation of paxillin, which facilitates vinculin recruitment into FAs to strengthen the connection between actin cytoskeleton and extracellular matrix, and thereby drive the FA maturation (Pasapera et al., 2010). Downregulation of ROCK1 protein level in two different cell lines, HT1080 and HeLa, induced by depletion of CA IX indicates that such ROCK1 mediated modulation of adhesion is a general and functionally important mechanism.

Multiple ROCK1 functions support the assumption that the ROCK1 downregulation caused by CA IX silencing can have a broader impact on adhesion-migration-invasion phenomena. In the previous paper, we documented a decreased expression of MMP9 in *CA9*-silenced cells (Radvak et al., 2013). Notably, reduction of ROCK1 level in these cells could be the mechanism responsible for MMP9 downregulation. The specific inhibitor of ROCK1 (Y27632) as well as RhoA/ROCK pathway inhibitor (simvastatin) can reduce the level of MMP9 mRNA (Turner et al., 2005). Since MMP9 plays an essential role in invadopodia formation and degradation of the basement membrane, its reduction in *CA9*-silenced cells augments the CA IX relevance in the adhesion/invasion process. In addition, CA IX has previously been shown to regulate the activity of MMP9 presumably through extracellular production of protons (Sansone et al., 2009). Indeed, transfectants expressing an ion-translocation defective mutant of pH regulator NHE1 (Na^+^/H^+^ exchanger) also show decreased gene expression of *MMP9* (Putney and Barber, 2004). This indicates that pHe and pHi-related effects of CA IX protein also contribute to changes in the gene expression profile in *CA9*-silenced cells.

Collagen IV is another gene downregulated in response to *CA9* mRNA silencing (Radvak et al., 2013). Both MMP9 and collagen IV are accumulated at the protruding lamellipodia of bronchial epithelial cells (Legrand et al., 1999). Collagen IV promotes migration by providing the substrate for attachment of advancing lamellipodia and MMP9 facilitates turnover and the release of FC by the cleavage of collagen and consequent matrix remodeling. Thus, the influence of CA IX depletion on collagen IV and MMP9 mRNA levels can at least in part explain its effect on FA inhibition.

We previously demonstrated that the catalytic domain is important for the role of CA IX in cell migration/invasion (Svastova et al., 2012). However, our present findings point to the N-terminal PG domain that appears to mediate CA IX effects on cell adhesion to the support especially in relation to the maturation of FC. The evidence is based on our observations of the reduced spreading of cells expressing the truncated form of CA IX that lacks the PG-domain as well as of cells expressing the full length CA IX protein that are treated with the PG domain-binding M75 antibody.

Among all known carbonic anhydrases, only CA IX contains this PG-like N-terminal extension of the extracellular catalytic domain, which displays 38% identity with a keratan sulfate attachment domain of the large aggregating proteoglycan aggrecan (Opavsky et al., 1996). Interestingly, comparative analysis of the core sequence (aa 62–93) of the PG domain containing the pentameric EEDLP repeat revealed even higher, 50% identity to the conserved hexapeptide sequence of the keratan sulfate-enriched region of aggrecan. This part of the aggrecan molecule exhibits a capacity to bind collagen and hyaluronan (Hedlund et al., 1999) and thus it is imaginable that the PG domain of CA IX can behave in a similar manner and bind ECM components.

In the phage display library, Zavada et al. (2000) identified the SASAPVS peptide competing with M75 antibody in binding to PG domain of CA IX. Our alignment analysis performed in BLAST (Basic Local Alignment Search Tool, NIH) showed 100% identity of this peptide with the stromal component mucin (Table 1). This is a very interesting finding as mucins are important ECM components contributing to cancer progression. Indeed, aberrant production of mucins is directly connected with the development of pancreatic, breast, colorectal and lung tumors (Hollingsworth and Swanson, 2004; Torres et al., 2012; Valque et al., 2012). Hollingsworth and Swanson (2004) suggest that tumors can use mucins to assemble the local microenvironment during invasion and metastasis in hostile conditions of hypoxia and acidosis.

**Table 1:** **BLAST analysis of peptide SASAPVS competing with M75 binding site in the PG domain of CA IX protein showing 100% identity with gastric mucin**.

**Alignment statistics for match**				
**Score**	**Expect**	**Identities**	**Positives**	**Gaps**
19.7 bits (39)	304	6/6 (100%)	6/6 (100%)	8
Competing peptide	2	ASAPVS	7	
		ASAPVS		
Mucin	2331	ASAPVS	2336	

The idea that CA IX potentially has the ability to bind mucin through its PG domain surely deserves further investigation, especially given the indirect evidence for this relationship provided in several published studies. Saarnio et al. (1998) were the first to notice that CA IX staining in colorectal carcinoma tissues showed the most intense signal in five of six adenocarcinomas with the mucinous component. Similar an observation was later described in ovarian tumors, in which the strongest immunohistochemical reactions detecting CA IX occurred in the borderline mucinous cystadenomas and mucinous cystadenocarcinomas (Hynninen et al., 2006). CA IX is also highly expressed in lung adenocarcinomas with mucinous component (Kon-No et al., 2006). Moreover, a direct association between CA IX and MUC1 protein expression was noted in non-small cell lung cancer (Giatromanolaki et al., 2001). The binding of CA IX to mucins may provide one of possible explanations for these findings.

Returning to the role of CA IX in FA, it is important to be aware that it strongly depends on the situation that leads either to adhesion and spreading or to migration. While the former process involves formation and maturation of FC, the latter requires dynamic remodeling of these contact structures. CA IX participates in FAs during spreading, but there is a role for this protein also in transient FC in migrating cells.

During cell migration, the actin reorganization and attachment of the leading edge to the substrate are the driving forces that regulate movement of the advancing lamellipodia (Pollard and Borisy, 2003; Zaidel-Bar et al., 2003). Nascent adhesions that assemble at the front of the growing lamellipodium are independent of myosin II (the important player in spreading) and are characterized by branched actin arrangement. The rate of assembly and turnover of these adhesions essentially depends on the actin polymerization coinciding with the actin severing activity (Choi et al., 2008). In migrating cells, CA IX colocalizes with paxillin directly in the nascent adhesions. Since these contacts in the leading edge exert transient forces to move the cell forward whilst only the maturation of FAs provides anchors to the substrate, our assumption is that it is the effect of the catalytic activity of CA IX on the acidification of the extracellular space that plays a role in nascent adhesion turnover.

There is increasing evidence that intracellular and extracellular pH gradient influences cell migration in many aspects (Cardone et al., 2005; Stock and Schwab, 2009; Martin et al., 2010). Changes in pHi induced by different cytokines and cellular processes affect protein conformations and macromolecular assemblies (Srivastava et al., 2007). We demonstrated that CA IX interacts with bicarbonate transporters in the lamellipodia of moving cells (Svastova et al., 2012). This interaction seems to be functionally active because coexpression of CA IX with NBC or AE1,2,3 maximizes the rate of bicarbonate transport across the plasma membrane (Morgan et al., 2007; Orlowski et al., 2012). Thus, bicarbonate ions produced by the enzymatic activity of CA IX can contribute to increased pHi in the leading edge in accordance with the established model of pH gradient both inside and around migrating cells.

Cofilin is considered to be one of the intracellular pH sensors which localizes to sites of intensive actin turnover (Srivastava et al., 2007). By severing F-actin it generates actin monomer pool, thus sustaining a high rate of filament elongation and membrane protrusion. The activity of cofilin coincides with pH gradient in migrating cells (Stock and Schwab, 2009). At higher pHi typical for the leading edge, deprotonation of cofilin releases its inhibitory binding to PI_(4,5)_P2 and leads to the acquisition of its active state (Frantz et al., 2008). Released cofilin then generates free-barbed ends of F-actin and promotes advancing lamellipodia. On the other hand, inactivation of cofilin activity stimulates the formation of actin stress fibers and inhibits cell migration (Toshima et al., 2001; Mouneimne et al., 2006).

Proton efflux and consequently increased pHi mediated by the pH regulator of migration NHE1 is necessary for the cofilin-dependent actin filament assembly in response to migratory stimuli (Frantz et al., 2008). CA IX also appears to participate in this process, since lamellipodia form predominantly in the CA IX-positive cells as soon as 15 min after the induction of migration in wounded monolayer (Svastova et al., 2012). Colocalization of CA IX with actin in the places of dynamic actin reorganization and its concurrence with paxillin in nascent adhesions in lamellipodia where CA IX directly interacts with bicarbonate transporters indicate that pH modulation is the method by which CA IX can contribute to actin dynamics and adhesion turnover during migration.

Interestingly, the generation of free barbed ends by cofilin activity defines the location of actin-based protrusions and initiates this process. It is documented that exactly the loss of cofilin binding to PI_(4,5)_P_2_, which is also regulated by pHi, is the key mechanism for cofilin activation in the leading edge of mammary carcinoma cells (Van Rheenen et al., 2007). In addition to cofilin, other proteins including talin, vinculin, and gelsolin display pH-dependence of actin binding properties, thus modulating FA remodeling and migration rates (Srivastava et al., 2008).

As we mentioned before, the turnover of FC in the lamellipodia and the rear end is the limiting process regulating migration rate. In the cell rear, a proton-sensitive TRPM7 channel (Ca^2+^ and Mg^2+^ permeant ion channel, transient receptor potential melastatin 7) regulates FA through calcium-dependent protease m-calpain inducing loss of adhesion via cleavage of the adaptor protein talin from integrin-cytoskeleton connection (Su et al., 2006; Stock and Schwab, 2009). In the cell front, i.e., in the lamellipodium, NHE1 as one of the most effective extruder of protons causing extracellular acidification, strongly modulates α2β1 integrin-dependent adhesion and migration of human melanoma cells (Stock et al., 2005). It is important to note that NHE1 and integrin receptors often accumulate at the leading edge of lamellipodia and thus NHE1 could generate acidic microdomains (Grinstein et al., 1993). The local pHe in close proximity to focal contact sites then influences the strength of FA and migration (Stock et al., 2005).

In summary, based on this work we propose that CA IX is a functional component of the FA process contributing to both maturation and dynamics of focal complexes depending on the situation that requires either cell spreading or migration. To accomplish this role, CA IX employs its extracellular catalytic domain that appears to be more relevant for the nascent adhesions during migration as well as its N-terminal PG-like domain that is rather required for the adhesion during spreading, although we cannot exclude the crosstalk of these domains in either situation. Indeed, *in vitro* data from the kinetics measurements of the recombinant enzymes suggest that the PG domain considerably increases the catalytic activity of CA IX and makes it the most active CA isoform (Hilvo et al., 2008).

In contrast to other CA isoenzymes that mostly occur in healthy tissues, CA IX is predominantly expressed in tumors in response to hypoxia. Hypoxia also stimulates other constituents of the migration machinery (including ion transporters and pH regulators, such as NHE1) (Cardone et al., 2005; Gatenby et al., 2007) and increases the ability of CA IX to regulate pH through the bicarbonate transport metabolon (Svastova et al., 2004; Morgan et al., 2007). Therefore, it is not surprising that it also participates in cell migration and invasion, thus contributing to the escape of tumor cells from the primary tumor mass in the pH-dependent manner. On the other hand, when tumor cells released to circulation reach the secondary site, they need to adhere and spread to initiate the growth of metastatic lesion, and this could be facilitated by CA IX-improved maturation of the FC.

Although these assumptions are based on experimental results obtained with cultured cells, they clearly have support from *in vivo* studies as found by data mining of the Oncomine and GEO databases (described in M&M). SPIA in nine independent clinical studies of several tumor types with at least 2.0-fold up-regulated transcription of *CA9* revealed the FA pathway as one of the most consistently activated pathways in these tumors (Table 2). Conclusions of this study implicate that it could be reasonable to use a combined treatment strategy against CA IX, targeting both catalytic and PG domains, thus maximizing the effect on CA IX action in cell adhesion-migration-invasion and thereby preventing tumor dissemination.

**Table 2:** **Cancer gene expression profiling studies with up-regulated CA9 (FC>2) related to significantly activated focal adhesion pathway as identified by SPIA algorithm**.

**Cancer type**	**Oncomine dataset**	**GEO ID**	Study type^*^	**SPIA—Focal adhesion (KEGG:04510)**	
				pGFDR^**^	Status^***^
Colorectal carcinoma progression	–	GSE1323	T/M	7.19E-02	Activated
Colorectal carcinoma progression	–	GSE2509	T/M	7.24E-02	Activated
Colorectal carcinoma	Skrzypczak Colorectal	GSE20916	N/T	8.11E-03	Activated
Clear cell renal carcinoma	Yusenko Renal	GSE11151	N/T	4.24E-07	Activated
Clear cell renal carcinoma	Lenburg Renal	GSE781	N/T	4.41E-02	Activated
Clear cell renal carcinoma	Gumz Renal	GSE6344	N/T	5.25E-06	Activated
Cervical squamous cell carcinoma	Scotto Cervix 2	GSE9750	N/T	3.48E-03	Activated
Esophageal carcinoma	Hu Esophagus	GSE20347	N/T	1.37E-06	Activated
Pancreatic carcinoma	PlaceStatePei Pancreas	GSE16515	N/T	6.45E-05	Activated

* Study type—T/M, primary tumor/metastasis; N/T, normal tissue/tumor.

** pGFDR—false discovery adjusted global probability testing of the hypothesis that the differentially expressed genes significantly perturb this pathway. The lower the pGFDR value is the lower the probability of observing this or a higher number of differentially expressed genes just by chance.

*** Status—indicates whether the pathway is activated or inhibited in the conditions of the study.

### Conflict of interest statement

The authors declare that the research was conducted in the absence of any commercial or financial relationships that could be construed as a potential conflict of interest.
